# One-year performance of posterior narrow diameter implants in hyperglycemic and normo-glycemic patients—a pilot study

**DOI:** 10.1007/s00784-021-03957-x

**Published:** 2021-05-03

**Authors:** Anton Friedmann, Marianna Winkler, Daniel Diehl, Mehmet Selim Yildiz, Hakan Bilhan

**Affiliations:** 1 Department of Periodontology, School of Dentistry, Faculty of Health, Witten/Herdecke University, Alfred-Herrhausen Str. 45, 58455 Witten, Germany; 2 Institute of Pharmacology and Toxicology, Center for Biomedical Education and Research (ZBAF), Faculty of Health, Witten/Herdecke University, Stockumer Straße 10, 58453 Witten, Germany; 3 Department of Periodontology, Faculty of Dentistry, Altınbaş University, Zuhuratbaba, İncirli Cd. No:11-A, 34147 Bakırköy, Istanbul, Turkey

**Keywords:** Marginal bone loss, Type 2 diabetes mellitus, Narrow diameter implants, SLActive surface, TiZr implants

## Abstract

**Objectives:**

The aim of the study was to compare the performance of narrow diameter implants in patients with uncontrolled diabetes mellitus type 2 (T2DM) and normo-glycemic individuals during the first 12 months after implant loading.

**Material and methods:**

In 16 T2DM patients with HbA1C > 6.5% (test group) and 16 normo-glycemic patients (HbA1C < 6.0%; control group), one to two narrow diameter tissue level implants were placed in the posterior maxilla or mandible. After 3-month lasting integration period, implants were loaded by fixed dentures. The clinical parameters probing depth (PD), bleeding on probing (BOP), attachment loss (CAL), recession and papilla bleeding index (PBI) were assessed manually at loading and after 12 months of function. The paired digital periapical radiographs were analyzed with regard to the change in marginal bone level (MBL) from baseline to 12 months’ control. The mean values calculated for both patient groups were statistically analyzed. The technical complications were recorded.

**Results:**

The T2DM group accounted 13 patients due to 3 dropouts. The overall implant survival rate after 12 months was 100%. The differences in means for the clinical parameters and the MBL were statistically non-significant between the T2DM and normo-glycemic patients for the short period of loaded function reported here. No technical complications were recorded.

**Conclusions:**

The study demonstrated an encouraging clinical outcome with narrow diameter implants in patients with uncontrolled T2DM compared to non-diabetics after 12 months post loading. For the short observation period, no biological and technical complications were reported regardless the glycemic status.

**Clinical relevance:**

Patients with HbA1C > 6.5% may benefit from the treatment with narrow diameter implants by avoiding complex surgical interventions with augmentation procedures.

**Trial registration:**

Clinicaltrials.gov: NCT04630691

**Supplementary Information:**

The online version contains supplementary material available at 10.1007/s00784-021-03957-x.

## Introduction

Type 2 diabetes mellitus (T2DM) is a widespread disease, which increasingly affects the society. It is described as a group of metabolic disorders which is characterized by high serum glycemic levels either due to insufficient insulin levels, defective function or both [1]. It is reported that patients with prediabetes and T2DM are associated with an increased risk of periodontal disease [2]. Hyperglycemia, being seen in uncontrolled T2DM, may be a potentially important factor in the development of biologic complications in dental implants. Prospective data on implant performance in diabetes patients are scarce. Most studies evaluating the effects of diabetes on implant success have studied patients with well-controlled diabetes. Recent studies indicated that dental implants may show a poorer outcome in high glycemic level patients with regard to probing pocket depth (PPD) and marginal bone loss (MBL) as compared with systemically healthy individuals [3, 4]. A recent 12-month follow-up meta-analysis found a significant increase in probing depth (PD), bleeding on probing (BOP) and marginal bone loss (MBL) in T2DM patients with controlled HbA1C when compared to healthy patients, pointing out that T2DM patients are likely to be at higher risk for peri-implant disease [5]. The authors concluded, however, that despite their findings, more long-term, well-controlled clinical studies are needed in order to establish a clearer opinion between peri-implant parameters and T2DM.

The number of patients undergoing restorative dental therapy using dental implants has grown significantly during the last decades [6]. The definition for an implant success described implants without any biological complications, technical complications or negative esthetic outcomes [7]. BOP, PD and MBL were considered as parameters suitable for monitoring biological complications at dental implants. An excess in marginal bone loss was considered a negative impact on biological as well as esthetic outcomes. For this reason, the maintenance of marginal bone level by the choice of implant geometry, specific surface characteristics and careful planning together with a minimally invasive surgical method became an ultimate task in implant therapy.

Narrow diameter implants were developed for sites with diminished ridge dimensions which result from numerous clinical reasons. This type of implant was approved successful for supplementing the posterior teeth in healthy patient cohorts, as reported in a systematic review [8]. A recent meta-analysis showed that the use of narrow diameter implants instead of regular diameter implants with bone augmentation procedures did not reveal differences in survival rates and marginal bone loss rate within the reported period [9]. A recent systematic review concluded non-significant difference in longevity and survival as for narrow and standard implants supporting single implant restorations, albeit the reduced-diameter implants more likely disclosed greater marginal bone loss [10]. The use of titanium-zirconium (TiZr) alloy implants for narrow implants has significantly increased biomechanical resistance, as shown in dynamic fatigue resistance tests, widening the indication range and making the use of narrow diameter implants in the posterior possible [11].

In uncontrolled T2DM patients who experienced tooth loss due to periodontal condition, narrow diameter implants might become an alternative option preventing invasive augmentation procedures and reducing the wound healing burden. The aim of this pilot clinical study was to compare clinical parameters and marginal bone level changes at NDI placed in the posterior maxillary and mandibular zones and loaded by fixed prosthesis in uncontrolled T2DM and normo-glycemic patients.

## Material and methods

The recruitment of participants was restricted to the patients from the Department of Periodontology and included individuals who were compliant with the supportive periodontal treatment (SPT) program after the course of active periodontal treatment. Thirty-two patients aging between 53 and 82, with a mean age of 67, participated in this pilot prospective clinical study (Table 1). Sixteen patients known to suffer from T2DM and diagnosed with an HbA1_C_ > 6.5% were considered as “uncontrolled” hyperglycemic and assigned for the test group, whereas 16 non-diabetic patients (HbA1C ≤ 6.0%) were allocated as controls. HbA1_C_ was assessed by the patient’s general practitioner, who submitted the results to our clinic. The ethics committee of Witten/Herdecke University approved the study protocol (108/2012), and all participants signed the informed consent. The study treatment modalities complied with the Declaration of Helsinki and fulfilled the Good Clinical Practice (GCP) criteria. The exclusion criteria were as follows:

- Immobility
- Periodontal surgery and/or antibiotic therapy within the last 6 months prior to baseline
- Pregnancy and lactation period
- Full Mouth Plaque Score (FMPS) > 25%
- Untreated periodontitis
- Smoking > 10 cigarettes/day
- Insufficient crestal width which affords an augmentation procedure even in the case of NDI
- Previously performed ridge augmentation procedure for a staged implant placement
- Permanent medication affecting blood perfusion rate and bone metabolism

**Table 1:** Patient demographics

	**All Groups**	**Test**	**Control**
**Patients (dropouts)**	32 (3)	16 (3)	16
**Mean age (range) Sex**	67	70 (53–87)	65 (53–84)
Male (%)	14 (48.3%)	8 (61.5%)	6 (37.5%)
Female (%)	15 (51.7%)	5 (38.5%)	10 (62.5%)
**Mean HbA1C (**±**SD)**	-	7.34(±0.73)	-
**Jaw**			
Maxilla	19	8	11
Mandibula	29	15	14
**Implant total**	48	23	25
**Implant length**			
8 mm	9	6	3
10 mm	23	10	13
2 mm	16	7	9
**Implant dropouts**	4	4	0

Each patient received one to maximum two NDI at an edentulous posterior region of either maxilla or mandible. Exclusively narrow diameter (3.3-mm) tissue level (RN TL) titanium-zirconium alloy implants (Roxolid®) with the SLActive® surface characteristic were used (Institut Straumann AG, CH). All implants were placed by two experienced periodontists, according to the instructions of the manufacturer regarding the osteotomy. The surgical approach was standardized: a mid-crestal incision in the edentulous area was combined with intrasulcular incisions in neighboring teeth while vertical releasing incisions were declined. A buccal and a lingual flap was minimally reflected to have a clear view to the crest. The placement was carried out under local anesthesia (Ultracain DS forte®—Sanofi-Aventis, Frankfurt, Germany) strictly following the standard transmucosal healing protocol in both the test and the control groups. The implants were planned to restore the site by either a single crown or a fixed partial denture (FPD). The screw or cementum retention was unrestricted by the protocol; however, all restorations used either SynOcta® or Variobase® abutments (Straumann®, Institut Straumann AG, CH). If two implants were placed, the most posterior one served as the study implant for this patient. Completing the surgery, all implants were radiographically documented using the parallel technique for periapical X-rays.

**Fig. 1:**
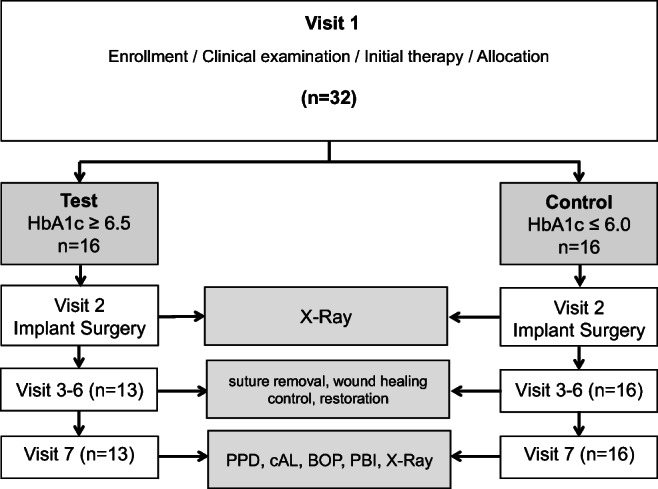
Flowchart of study protocol. Visit 3 = 3 days, visit 4 = 10 days, visit 5 = 1 month, visit 6 = 3 months, visit 7 = 12 months. PPD probing pocket depth, cAL clinical attachment loss, BOP bleeding on probing, PBI papilla bleeding index

The post-op regimen included the patient’s instruction to abstain from mechanical plaque control in the treated area for 1 week and to use chlorhexidine (Chlorhexamed GlaxoSmithKline Consumer Healthcare GmbH & Co. KG, Munich, Germany) mouth rinse (0.2%) twice a day instead. The administration of systemic antibiotics was restricted to individual needs. There was no prescribing policy by protocol, and analgesic medication (Ibuprofen 600 mg/3× daily) on demand was recommended. A follow-up visit after 3 days was scheduled, and sutures were removed after 7–10 days. After 12 weeks, the next follow-up was to evaluate the osseointegration before starting the reconstruction. At 1 year (Visit 7), the reported measurements were obtained (Fig. 1).

Chipping of the porcelain coating, screw loosening, de-cementation, fracture of any component or other maintenance requirements were considered technical complications and recorded during the observation period.

### Assessment of clinical parameters

The peri-implant PD, CAL and recession were estimated by gentle probing with a PCP-11 probe (Hu-Friedy, Tuttlingen, Germany) at 4 sites per implant. Additionally, the PPD of the adjacent tooth at 4 sites of the tooth was assessed, representing a native reference for each study implant. The measurements were carried out immediately after loading (visit 3) and 12 months after implant surgery (visit 7) on both the integrated implants and teeth. Furthermore, the bleeding on probing (BOP) and papilla bleeding index (PBI) on the buccal aspect were investigated at visit 7. Figure 1 displayed the study protocol as a flow chart.

### Radiography and measurement of the marginal bone level

**Fig. 2:**
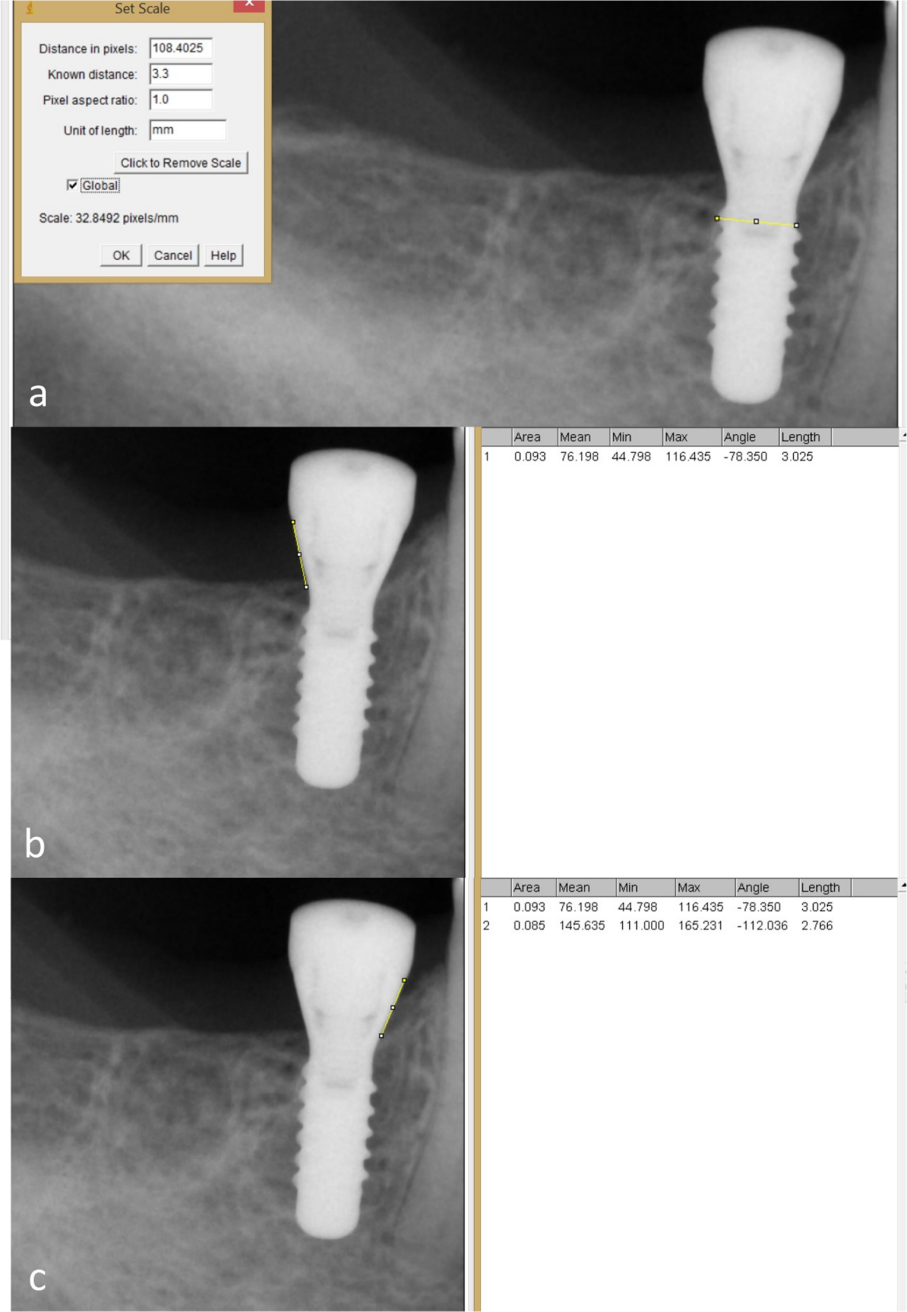
Radiographic images disclosing the principle for MBL assessment at visit 7. **a** Adjustment of distortion coefficient. **b** Distal aspect of the implant. **c** Mesial aspect of the implant. For further measurements, the mean values from distal and mesial aspects were calculated

The digital radiograph from immediately after implantation (“initial”) and 1 year later (“12 months”) using the parallel technique and a conventional sensor holder (Sidexis, Dentsply Sirona, Bensheim, Germany) was available. Each pair of radiographs was accounted for interpatient calibration by estimating the distortion coefficient adjusting the images to the given implant diameter (Fig. 2a). The distance between single threads of each implant projected on the standard monitor served for calculation of the distortion coefficient in the vertical dimension [12]. The same investigator (M.S.Y.) utilizing ImageJ2 software [13] conducted all marginal bone level (MBL) assessments. To establish a reference level for assessment of vertical bone dimension, two landmarks were determined on each radiograph. The landmarks indicated the most coronal point of the crestal bone in contact with the implant at the initiation of the rough surface on the mesial and distal aspects on the first, the “initial” radiograph. A perpendicular line along the implant axis was drawn between these landmarks, and the distance was calculated in millimeter (Fig. 2b,c). The measurement was repeated on visit 7 (“12 months”) radiograph estimating again the reference lines according to the same principle for detection of change in crestal bone position.

### Statistical analysis

The mean and standard deviation were calculated for quantitative variables. All statistical analyses were performed by Prism 8 (GraphPad Software, San Diego, CA, USA). The statistical evaluation included the Kolmogorov-Smirnov testing for the Gaussian distribution of raw data. All variables were subject to normal distribution; therefore, parametric tests were used for further analysis. For clinical parameters, differences between study groups were evaluated by Student’s *t* test. For group comparisons of marginal bone level, a delta value (ΔMBL = MBL_12months_ − MBL_3months_) was calculated and evaluated by Student’s *t* test. The significance level was set at *p* = 0.050. Due to the pilot character of the study, power analysis was performed a posteriori. Accordingly, the effect size *d* was calculated for the comparisons of PD and MBL utilizing the mean and standard deviations in G*Power [14]. After that, a post hoc power analysis was performed.

## Results

In 32 patients, a total of 48 osseointegrated implants qualified for the prosthetic loading at the 3-month pre-load analysis. Some patients discontinued treatment after this inspection unintentionally; thus, the T2DM group experienced a decline by 3 patient dropouts with a total of 4 implants, respectively. The rationale for dropouts was strongly individual. One patient missed the impression-taking visit for unknown reasons. The second patient was identified as type 1 diabetes mellitus who received both implants placed in the anterior section of an edentulous maxilla and thereby double violating the inclusion criteria. The third one underwent a stomach surgery with a fatal consequence before the implant was restored. Thus, 13 NDI study implants from the T2DM group remained to compare to 16 NDI study implants from the normo-glycemic group after completing the restorative phase (Table 1). Accounting for the dropouts, the mean statistical HbA1c value was calculated with 7.34% for the diabetic group.

All restored implants were under functional load after 12 months, resulting in an overall survival rate of 100% for both groups, respectively. Three single crowns in two patients were screw retained; all other crowns or bridge frameworks were cemented using glass-ionomer luting cementum (Ketac Cem, ESPE, Germany). There were no complaints; no biological or technical complications or adverse events related to the implant treatment were reported by the patients after 1 year of function regardless the glycemic status.

**Fig. 3:**
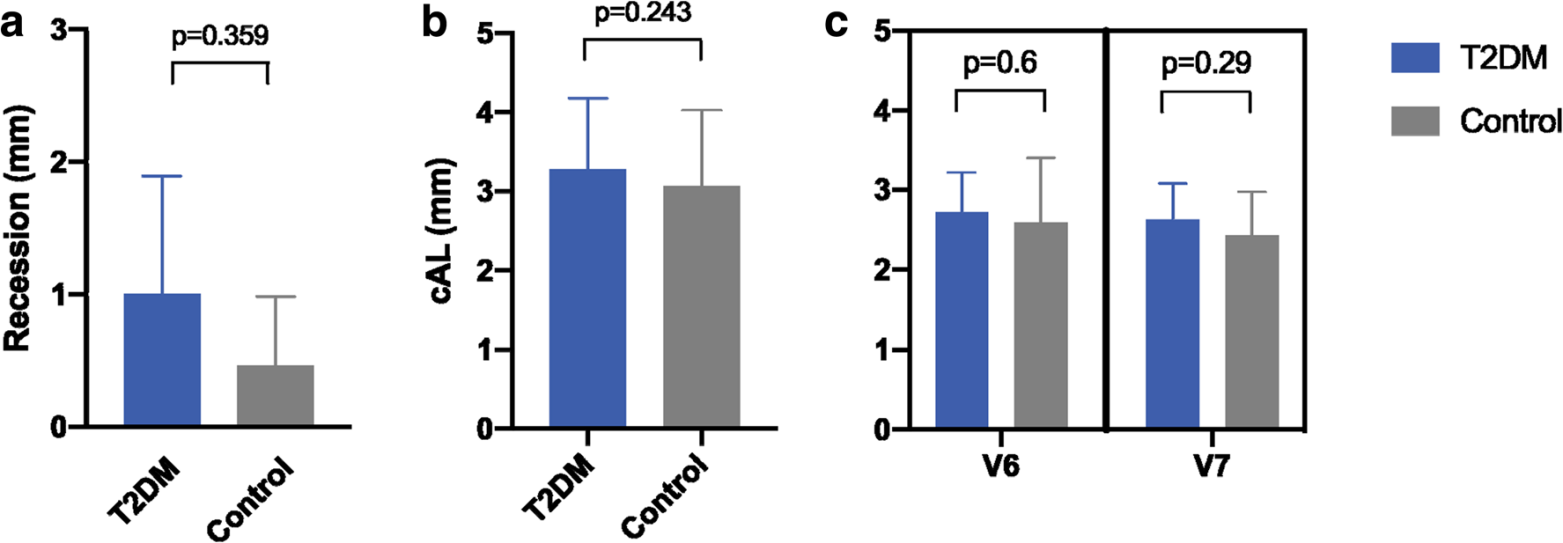
Results for statistical evaluation of clinical measurements. **a** Clinical attachment loss. **b** Recession. **c** Probing pocket depth

**Fig. 4:**
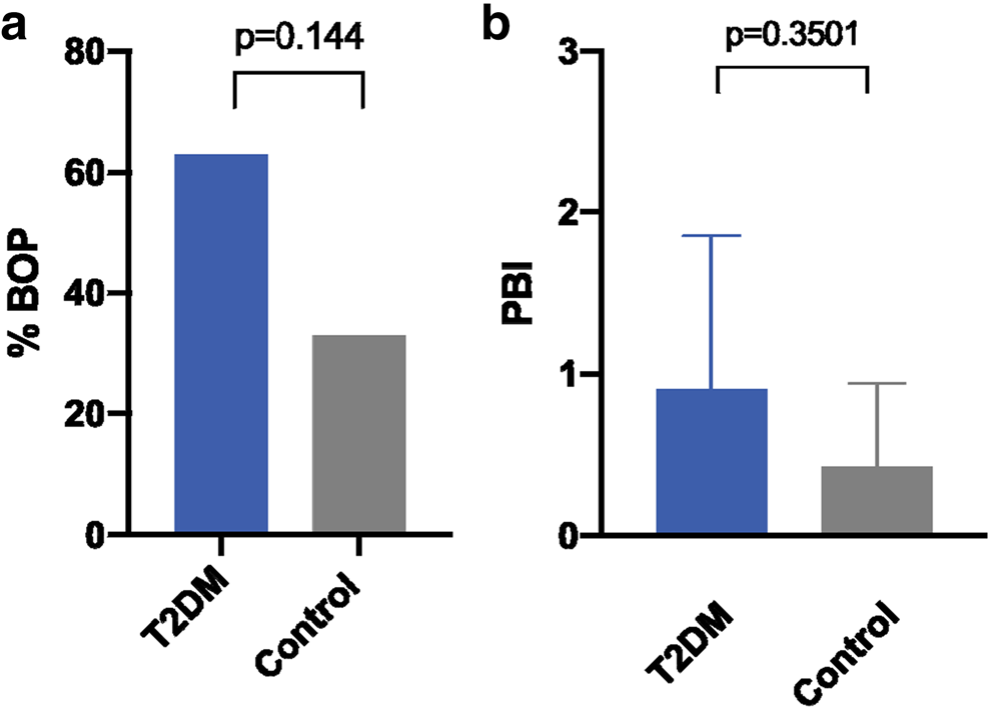
Descriptive statistics for **a** BOP and **b** papilla bleeding index (PBI)

The mean peri-implant PD at 3 months was measured at 2.6 ± 0.8 mm for the normo-glycemic group, whereas the T2DM group exhibited a mean PD of 2.7 ± 0.5 mm before loading. After 12 months, the mean values changed towards 2.4 ± 0.5 mm and 2.6 ± 0.4 mm in the groups, respectively (Fig. 3c). Thus, the mean values for the clinical parameters assessed at 3 and 12-month visit revealed statistically non-significant differences (*p* = 0.6 and *p* = 0.29, respectively). The BOP index, however, appeared slightly increased in the T2DM compared to the control group (63% to 54%) at visit 7, whereas the PBI remained indifferent (*p* = 0.351) in both groups (Fig. 4a,b).

**Fig. 5:**
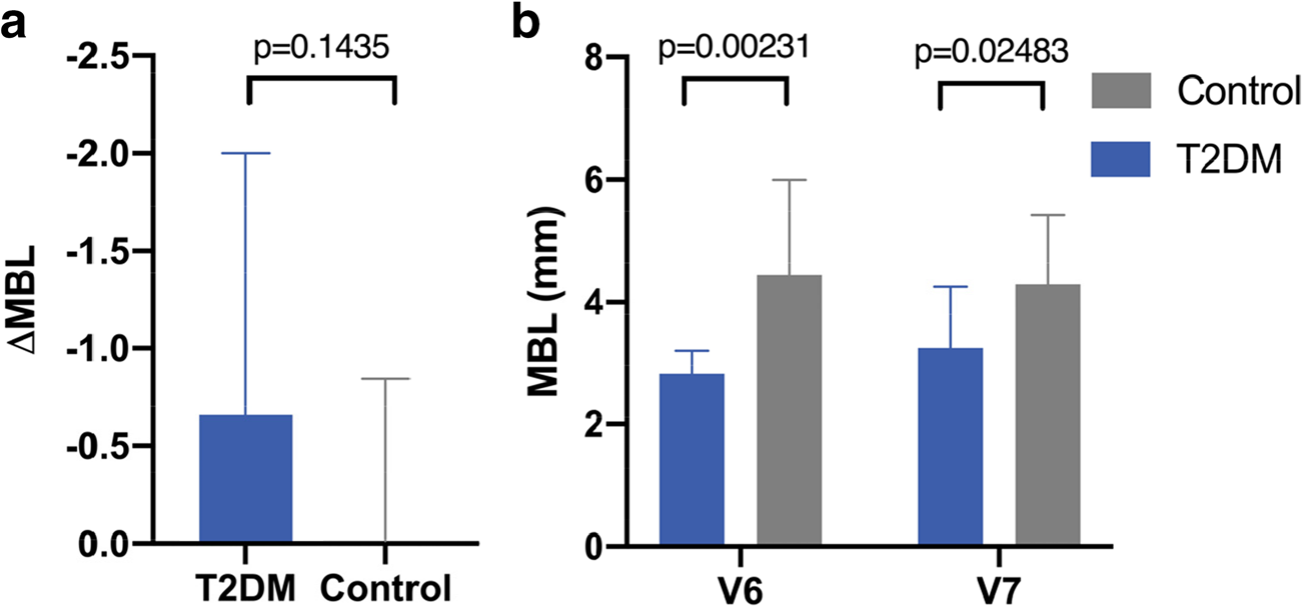
Results from radiographic evaluation of marginal bone loss (MBL). **a** Comparison of mean ΔMBL (MBL_12months_ − MBL_3months_). **b** Descriptive statistics of mean MBL measurements at visits 6 and 7

The radiographic analysis revealed a non-significant change of the marginal bone level (MBL) for both study groups. The ΔMBL was calculated with −0.014 mm for non-diabetic and −0.659 mm for the T2DM patients. The 12-month comparison between both groups disclosed non-significant difference (*p* = 0.144) in ΔMBLs (Fig. 5a,b).

**Fig. 6:**
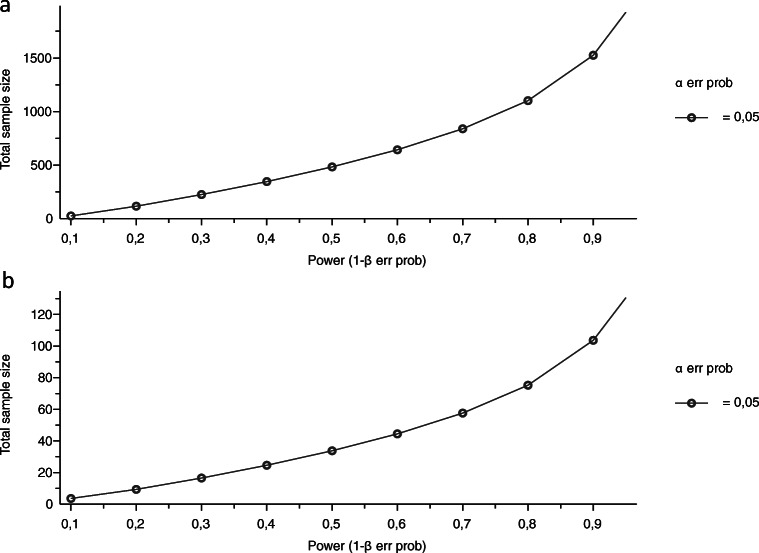
Plots for necessary sample size in **a** PD measurements and **b** MBL measurements

According to the acquired data, the effect size *d*, as calculated by G*Power, was 0.25 for the PD measurements and 0.57 for the mean ΔMBL values. Thus, the post hoc power analysis revealed a power (1 − *β* error probability) of 0.15 for PD measurements and 0.42 for MBL measurements (Fig. 6, Table 2).

**Table 2:** Summary of all measurements taken during the study, including *p* values

		**Test**	**Control**	***p***	**Power (1 −** 휷 **error)**
Probing depth in mm (mean ± SD)	3 months	2.7 ± 0.5	2.6 ± 0.8	0.912	0.15
	12 months	2.6 ± 0.4	2.4 ± 0.5		
Recession in mm (mean ± SD)		1.0 ± 0.89	0.47 ± 0.52	0.359	
∆MBL (mm)	MBL_visit 6_ − MBL_visit 7_	0.659 ± 1.34	0.014 ± 0.83	0.144	0.42
BOP (% per group)		63%	33%	0.144	
PBI (mean ± SD)		0.9 ± 0.94	0.43 ± 0.51	0.351	

## Discussion

The study enrolled 16 T2DM patients with an HbA1c level > 6.5% whose mean level exceeded the 7.3% threshold. The type 2 diabetes mellitus (T2DM) diagnosis in patients with an HbA1c level > 6.5% was confirmed by the recent ADA definition [15]. It was claimed that HbA1c levels around 7% may reduce microvascular complications and decrease the macrovascular damage in the long term [16]. Accounting for the 13 retained in the study of T2DM patients, the interpretation of the mean value implicated that several patients were insufficiently controlling the hyperglycemia.

The population-based “SHIP” study from Germany revealed that patients suffering from a poorly controlled diabetes were associated with much greater attachment loss and tooth loss numbers than pre-diabetic or well-controlled diabetic patients [17]. Based on the observations in a recent overview of systematic reviews, the authors concluded that there was no effect of diabetes on the survival rate of implants, but on MBL, affecting thereby the osseointegration [18]. Correspondingly, another recent systematic review concluded that there was no difference in longevity and survival rates between narrow and standard implants supporting single implants [10].

The definition of a successful implant described an implant without any biological complications, technical complications or negative esthetic outcomes [7]. Bleeding on probing (BOP), peri-implant probing depth (PD) and radiographical marginal bone loss (MBL) were recommended to assess biological implant success [19]. As indicated by several longitudinal studies, implant surface characteristics, meticulous case planning and minimally invasive surgical techniques had intense input on the outcome [20]. Among other clinical parameters, the BOP values were non-significantly different between both groups at 1-year visit, appearing upregulated for the total study population. However, neither the clinical parameters nor the X-ray analysis was indicating any progressive loss in bone attachment that occurred between visit 3 and visit 7 (Table 2). Any potential of cementum retained supra-structures to cause biological complication was dismissed as implant placement strictly respected the SP design of the implant shoulder positioning it explicitly epi-gingivally. Thus, the distance between the cemented crown margin and the most crestal bone-to-implant contact equaled consistently almost 2 mm.

The tolerance for the initial MBL levels in generally healthy population after the first year of implantation oscillated from almost non-detectable to a level of 1.0–1.2 mm according to either rather recent or older studies [21–24]. Although dental implants are considered successful treatment modality with high survival and success rates, certain systemic conditions such as T2DM may endanger the clinical outcome [25]. All patients from the study remained compliant with the stringent SPT regimen using at least twice a year the services proposed by the dental hygienist.

Narrow diameter implants (NDI) were introduced for sites with spatial limitations and/or reduced ridge dimensions; patients enrolled into the study disclosed the diminished width of alveolar ridge at the edentulous area of interest.

A recent systematic review and meta-analysis of narrow diameter implants (NDI) compared to standard diameter implants (SDI) revealed widespread use of the NDI. The implants with a diameter of 3.3 to 3.5 mm (category 3) were supporting both the anterior and posterior restorations, respectively. However, the authors claimed the lack of long-term data and reported biological and technical complications for this NDI group [26]. Shi et al. reported an 8-year retrospective analysis of 98 NDI placed in both the premolar and molar areas for either a single implant supported or splinted restorations [27]. The overall implant survival rate was 96.9% at the implant and 97% at the patient level. The NDI design in their study matched the implant type used in our investigation by far with a difference in the height of the machined portion of the implant collar. Shi et al. used a 2.7-mm-long supracrestal part, whereas 1.8 mm supracrestal configuration was used for this study. A recent clinical trial compared the NDI to regular diameter implants (RDI) randomly placed in a posterior edentulous area at the maxilla and the mandible in 22 patients [28]. The authors reported non-significant difference in the marginal bone level assessed at both implant types after 1 and 3 years of function. These studies recruited explicitly healthy patients for the evaluation of NDI performance in posterior areas.

The type of NDI used in this study was the TiZr alloy with the SLActive® surface characteristic (Roxolid® by Straumann®, Institut Straumann AG, CH) and tissue level neck configuration. The development of special alloy compositions such as the TiZr made implants more resistant to mechanical failures. The use of TiZr alloys for narrow implants significantly increased biomechanical resistance, as dynamic fatigue resistance tests demonstrated, justifying the use of narrow diameter implants in the posterior area [29]. This fact may have contributed to the absence of any technical complications during the reported short period of function.

A 10-year retrospective observational study estimated 96.9% and 97% overall survival rates of the NDI used in the posterior jaw areas for supplementing either premolar or molar teeth supporting as single as splinted restorations [27]. A recent meta-analysis showed that the use of narrow diameter implants, instead of bone augmentation procedures with regular diameter implants, did not show differences in survival and marginal bone loss in the short term and the middle term [9]. A successful substitution of posterior teeth by NDI-supported reconstructions was reported by a systematic review for systemically healthy patient cohorts [8]. Titanium-zirconia (TiZr) NDIs presented similar success rates and similar peri-implant bone level change to commercially pure titanium implants [30]. The narrow diameter TiZr implants placed in the anterior area showed a mean bone loss of 0.3 ± 0.5 mm 1 year after placement in systemically healthy population [31]. The implants used in their study had bone level shape without the machined collar such as the tissue level implants. The latter used together with bone level implants revealed a mean MBL of 0.71 ± 0.58 mm and 1.55 ± 0.46 mm, respectively assessed in a clinical trial 2 years after prosthetic loading [32]. A narrative review pointed out that the tissue level implant was positively correlated with crestal bone level maintenance as the distance between bone crest and the margin of prosthetic restoration automatically exceeds 1.5 mm, a factor listed as one of the prerogatives against the onset of peri-implant lesions [33].

One RCT concluded that the outcome after using two implant surface characteristics in type 2 diabetic patients with relatively poor glycemic control missed to discriminate SLA from chemically modified SLActive® implants [34]. However, the results of an animal study indicated the negative effect of untreated diabetes mellitus on early osseointegration of dental implants showing at the same time an accelerated osseointegration for the modified SLA® surface (SLActive®) [35]. Once the critical period of the transition from primary stability to secondary stability [36] was passed, the bone healing and an optimal marginal bone level maintenance have optimal premises. Thus, a bioactive surface appeared beneficial for patients with compromised healing mechanisms.

This study showed encouraging clinical outcome for NDI in patients with poorly controlled T2DM and non-diabetics after 1 year of function. The peri-implant tissues and implant-borne restorations disclosed similar biological response and function for the NDIs in hyperglycemic as in normo-glycemic patients. These short-term observations indicated that the minimally invasive approach and the prevention of augmentation procedures resulted in an uneventful implant integration and initial function regardless the glycemic condition if using the NDI for support of single crowns or PFDs in the posterior edentulous areas.

## Conclusions

In terms of short-term implant success and implant survival, there were statistically non-significant differences between normo-glycemic and diabetic patients after a minimal invasive surgery was applied for implant placement eliding any augmentation procedure.

## Supplementary Information

ESM 1 (DOC 99 kb)

## References

[1] Zimmet P, Alberti KG, Magliano DJ, Bennett PH (2016). Diabetes mellitus statistics on prevalence and mortality: facts and fallacies. Nat Rev Endocrinol.

[2] Lamster IB, Cheng B, Burkett S, Lalla E (2014). Periodontal findings in individuals with newly identified pre-diabetes or diabetes mellitus. J Clin Periodontol.

[3] Alasqah MN, Alrabiah M, Al-Aali KA, Mokeem SA, Binmahfooz AM, ArRejaie AS, Abduljabbar T (2018). Peri-implant soft tissue status and crestal bone levels around adjacent implants placed in patients with and without type-2 diabetes mellitus: 6 years follow-up results. Clin Implant Dent Relat Res.

[4] Al-Sowygh ZH, Ghani SMA, Sergis K, Vohra F, Akram Z (2018). Peri-implant conditions and levels of advanced glycation end products among patients with different glycemic control. Clin Implant Dent Relat Res.

[5] Lagunov VL, Sun J, George R (2019). Evaluation of biologic implant success parameters in type 2 diabetic glycemic control patients versus health patients: a meta-analysis. J Investig Clin Dent.

[6] Armas J, Culshaw S, Savarrio L (2013). Treatment of peri-implant diseases: a review of the literature and protocol proposal. Dent Update.

[7] Lang NP, Pun L, Lau KY, Li KY, Wong M (2012). A systematic review on survival and success rates of implants placed immediately into fresh extraction sockets after at least 1 year. Clin Oral Implants Res.

[8] Klein MO, Schiegnitz E, Al-Nawas B (2014). Systematic review on success of narrow-diameter dental implants. Int J Oral Maxillofac Implants.

[9] Ma M, Qi M, Zhang D, Liu H (2019). The clinical performance of narrow diameter implants versus regular diameter implants: a systematic review and meta-analysis. J Oral Implantol.

[10] Telles LH, Portella FF, Rivaldo EG (2019). Longevity and marginal bone loss of narrow-diameter implants supporting single crowns: a systematic review. PLoS One.

[11] Kobayashi E, Matsumoto S, Doi H, Yoneyama T, Hamanaka H (1995). Mechanical properties of the binary titanium-zirconium alloys and their potential for biomedical materials. J Biomed Mater Res.

[12] Friedmann A, Meskeleviciene V, Yildiz MS, Götz W, Park J-C, Fischer KR (2020). Open healing of contained and non-contained extraction sockets covered with a ribose cross-linked collagen membrane: a pilot study. J Periodontal Implant Sci.

[13] Rueden CT, Schindelin J, Hiner MC, DeZonia BE, Walter AE, Arena ET, Eliceiri KW (2017). ImageJ2: ImageJ for the next generation of scientific image data. BMC Bioinformatics.

[14] Faul F, Erdfelder E, Lang AG, Buchner A (2007). G*Power 3: a flexible statistical power analysis program for the social, behavioral, and biomedical sciences. Behav Res Methods.

[15] American Diabetes A (2014). Diagnosis and classification of diabetes mellitus. Diabetes Care.

[16] American Diabetes A (2014) Executive summary: standards of medical care in diabetes--2014. Diabetes Care 37:S5–S13 10.2337/dc14-S005 24357214

[17] Kowall B, Holtfreter B, Volzke H, Schipf S, Mundt T, Rathmann W, Kocher T (2015). Pre-diabetes and well-controlled diabetes are not associated with periodontal disease: the SHIP Trend Study. J Clin Periodontol.

[18] Souto-Maior JR, Pellizzer EP, de Luna Gomes JM, Dds CAAL, Dds JFSJ, Vasconcelos BCDE, de Moraes SLD (2019). Influence of diabetes on the survival rate and marginal bone loss of dental implants: an overview of systematic reviews. J Oral Implantol.

[19] Buser D, Weber HP, Lang NP (1990). Tissue integration of non-submerged implants. 1-year results of a prospective study with 100 ITI hollow-cylinder and hollow-screw implants. Clin Oral Implants Res.

[20] Chappuis V, Buser R, Bragger U, Bornstein MM, Salvi GE, Buser D (2013). Long-term outcomes of dental implants with a titanium plasma-sprayed surface: a 20-year prospective case series study in partially edentulous patients. Clin Implant Dent Relat Res.

[21] Albrektsson T, Buser D, Sennerby L (2012). On crestal/marginal bone loss around dental implants. Int J Prosthodont.

[22] Goiato MC, Pellizzer EP, da Silva EV, Bonatto LR, dos Santos DM (2015). Is the internal connection more efficient than external connection in mechanical, biological, and esthetical point of views? A systematic review. Oral Maxillofac Surg.

[23] Lombardi T, Berton F, Salgarello S, Barbalonga E, Rapani A, Piovesana F, Gregorio C, Barbati G, Di Lenarda R, Stacchi C (2019). Factors influencing early marginal bone loss around dental implants positioned subcrestally: a multicenter prospective clinical study. J Clin Med.

[24] Pan YH, Lin HK, Lin JC, Hsu YS, Wu YF, Salamanca E, Chang WJ (2019). Evaluation of the peri-implant bone level around platform-switched dental implants: a retrospective 3-year radiographic study. Int J Environ Res Public Health.

[25] Jung RE, Zembic A, Pjetursson BE, Zwahlen M, Thoma DS (2012). Systematic review of the survival rate and the incidence of biological, technical, and aesthetic complications of single crowns on implants reported in longitudinal studies with a mean follow-up of 5 years. Clin Oral Implants Res.

[26] Schiegnitz E, Al-Nawas B (2018). Narrow-diameter implants: a systematic review and meta-analysis. Clin Oral Implants Res.

[27] Shi JY, Xu FY, Zhuang LF, Gu YX, Qiao SC, Lai HC (2018). Long-term outcomes of narrow diameter implants in posterior jaws: a retrospective study with at least 8-year follow-up. Clin Oral Implants Res.

[28] de Souza AB, Sukekava F, Tolentino L, Cesar-Neto JB, Garcez-Filho J, Araujo MG (2018). Narrow- and regular-diameter implants in the posterior region of the jaws to support single crowns: a 3-year split-mouth randomized clinical trial. Clin Oral Implants Res.

[29] Tolentino L, Sukekava F, Garcez-Filho J, Tormena M, Lima LA, Araújo MG (2016). One-year follow-up of titanium/zirconium alloy X commercially pure titanium narrow-diameter implants placed in the molar region of the mandible: a randomized controlled trial. Clin Oral Implants Res.

[30] Iegami CM, Uehara PN, Sesma N, Pannuti CM, Tortamano Neto P, Mukai MK (2017). Survival rate of titanium-zirconium narrow diameter dental implants versus commercially pure titanium narrow diameter dental implants: a systematic review. Clin Implant Dent Relat Res.

[31] Al-Nawas B, Domagala P, Fragola G, Freiberger P, Ortiz-Vigón A, Rousseau P, Tondela J (2015). A prospective noninterventional study to evaluate survival and success of reduced diameter implants made from titanium-zirconium alloy. J Oral Implantol.

[32] Cabrera-Domínguez JJ, Castellanos-Cosano L, Torres-Lagares D, Pérez-Fierro M, Machuca-Portillo G (2020). Clinical performance of titanium-zirconium implants with a hydrophilic surface in patients with controlled type 2 diabetes mellitus: 2-year results from a prospective case-control clinical study. Clin Oral Investig.

[33] Heitz-Mayfield LJA, Heitz F, Lang NP (2020). Implant disease risk assessment IDRA—a tool for preventing peri-implant disease. Clin Oral Implants Res.

[34] Khandelwal N, Oates TW, Vargas A, Alexander PP, Schoolfield JD, Alex McMahan C (2013). Conventional SLA and chemically modified SLA implants in patients with poorly controlled type 2 diabetes mellitus—a randomized controlled trial. Clin Oral Implants Res.

[35] Schlegel KA, Prechtl C, Möst T, Seidl C, Lutz R, von Wilmowsky C (2013). Osseointegration of SLActive implants in diabetic pigs. Clin Oral Implants Res.

[36] Barewal RM, Oates TW, Meredith N, Cochran DL (2003). Resonance frequency measurement of implant stability in vivo on implants with a sandblasted and acid-etched surface. Int J Oral Maxillofac Implants.

